# Dataset of endometrial blood flow from pregnant and non-pregnant mares on day 7 and 8 post-ovulation

**DOI:** 10.1016/j.dib.2020.105616

**Published:** 2020-04-25

**Authors:** Pilar Nieto-Olmedo, Gemma Gaitskell-Phillips, Francisco E Martín-Cano, Jose Manuel Ortiz-Rodríguez, Fernando J Peña, Cristina Ortega-Ferrusola

**Affiliations:** a CEFIVA-Centro de Fertilización In vitro de Asturias, Spain; b Laboratory of Equine Reproduction and Equine Spermatology, Veterinary Teaching Hospital, University of Extremadura, Cáceres, Spain

**Keywords:** Uterine blood flow, Endometrial angiogenesis, Pregnant mare, Doppler ultrasonography, Embryo transfer, Barren mare, Pregnancy diagnosis mare

## Abstract

This article provides the dataset for the use of power Doppler ultrasound to assess the equine uterus from the recent research article titled “Power Doppler can detect the presence of 7-8 days conceptuses prior to flushing in an equine embryo transfer program”(1). The vascularization of the endometrium was objectively assessed in mares by quantification of pixels in bitmap format (BMP) using computer assisted analysis of images. Fifty-two mares were examined on days 7 (26 mares) and 8 (26 mares) post-ovulation prior to performing flushing procedures for embryo recovery. Receiver operating characteristic (ROC) curves and Youden's J statistics were used to evaluate the value of the suggested variable in terms of its diagnostic value for identification of early pregnancy and to establish cut-off values allowing differentiation between pregnant and non-pregnant mares on days 7 and 8 post-ovulation.

Specifications table

**Table:** 

Subject	Veterinary Medicine
Specific subject area	Diagnostic imaging using Doppler ultrasound for early detection of pregnancy in mares
Type of data	Table
	Figure
How data were acquired	Ultrasonographic examination was performed using MyLabFive Vet equipment (Esaote S.p.A, Genova, Italy) and a linear 5–7.5 MHz probe. Rectal ultrasound examination was performed and the ultrasound probe was positioned transversely in the middle point of each uterine horn. Two still images were obtained per horn from the ultrasound examination. Signals for blood flow in all endometrial, myometrial and perimetrial vessels were visualized using Power flow modes and blood flow was measured in pixels.
	Still Doppler images were assessed using computer analysis with ImageJ v1.48 software (National Institute of Health, USA). ImageJ is a Java-based image processing program which is capable of calculating pixel value statistics for user-defined selections.
Data format	Raw
	Analyzed
Parameters for data collection	Fifty-two mares (Pure Bred Spanish horses), none of which showed signs of uterine pathology were used, with an age range of between 2 and 18 years old. Endometrial blood flow was evaluated, and embryo recovery was subsequently performed on days 7 and 8 post-ovulation. Mares were all sedated with romifidine (0.02 mg/kg) (Sedivet®, Barcelona, Spain), before ultrasonographic assessments were commenced.
Description of data collection	Rectal ultrasound examination was performed, and the ultrasound probe was positioned transversely in the middle point of each uterine horn. Pixel quantification was used to accurately and objectively assess the degree of uterine vascular perfusion. Blind analysis was performed on a total of 208 images (four images per mare) using computer analysis with ImageJ v1.48 software (National Institute of Health, USA). Detection of blood vessels was carried out on the images with application of a color threshold which was restricted to the uterine horn under examination.
Data source location	Institution: Faculty of Veterinary Medicine-University of Extremadura
	City/Town/Region: Cáceres, Extremadura
	Country: Spain
Data accessibility	With the article
Related research article	Pilar Nieto-Olmedo^a^; Francisco E Martín-Cano^b^; Gemma Gaitskell-Phillips ^a^; Jose Manuel Ortiz-Rodríguez ^a^; Fernando J Peña ^a^; Cristina Ortega-Ferrusola ^a^
	Power Doppler can detect the presence of 7–8 day conceptuses prior to flushing in an equine embryo transfer program.
	Theriogenology 145, 1-9.
	https://doi.org/10.1016/j.theriogenology.2020.01.015[1]

## Value of the data

• These data provide useful information concerning clinical applicability of power doppler ultrasound for discrimination between pregnant and non-pregnant mares on days 7 and 8 post-ovulation before performing embryo recovery.
• These data could be beneficial in clinical practice. The technique could be used in routine clinical practice by veterinarians in order to maximize embryo recovery rates from donor mares. Cut-off values established using this data can be used to predict pregnancy diagnosis prior to embryo collection.
• In addition, these data could be useful for further research into the study of early embryonic death and the changes which occur in uterine tissues immediately prior to it.
• The data may also prove useful with regard to development of strategies for implementation of timely preventative treatments for mares which are known to have suffered from early embryonic death in the past and have been unable to maintain pregnancies.
• These data can be used as a basis for the development of further experiments into the changes which occur to uterine blood flow after entry of the equine embryo into the uterus which could lead to further development of the technique as a diagnostic complement for early pregnancy diagnosis with future potential to avoid unnecessary flushing procedures in non-pregnant mares.
• Both the raw and analyzed data in pixels are presented in this article. These data could be exploited in order to train veterinarians who wish to learn how to perform the technique and used as a reference to ensure they are performing the procedure correctly. The raw data highlights the extent of variation that can be expected from measurements of uterine blood flow via Doppler images.

## 1 Data Description

This dataset provides a complete set of measurements from images showing blood flow to the equine uterus in the mare at 7 and 8 days post-ovulation which were obtained using power Doppler ultrasound. Quantification of pixels was used to objectively assess each image whilst in bitmap format (BMP) aided by computer assisted image analysis.

The dataset is shown in the following tables and figures:

Fig. 1, Fig. 2.

**Fig. 1:**
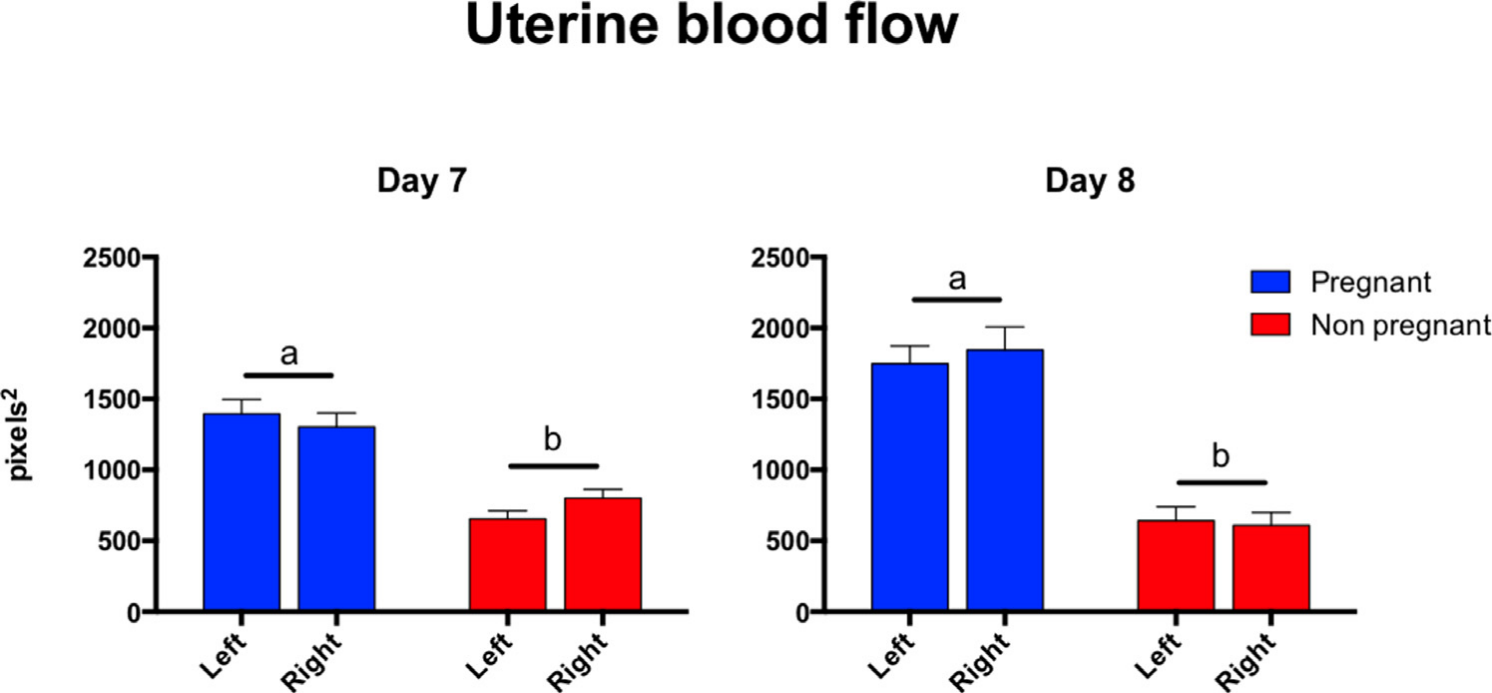
This graph represents the differences in endometrial blood flow between pregnant and non-pregnant mares on days 7 and 8 post-ovulation. The results are expressed in pixels as a mean ± SD. There were significant differences detected between pregnant and non-pregnant mares (p < 0.001). Vascularization between left and right horns showed no differences in either group.

**Fig. 2:**
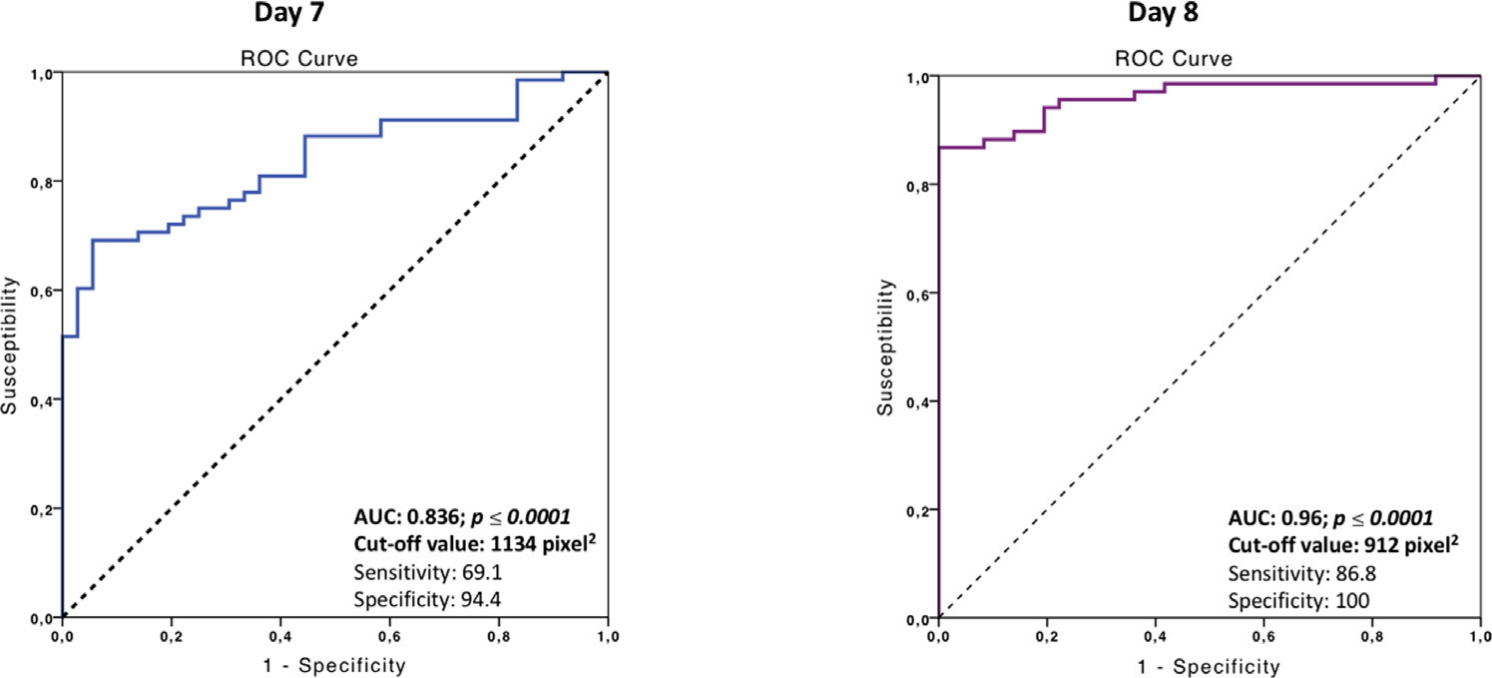
Receiver operating characteristic (ROC) curves for the blood flow area parameter (pixels) in mares on days 7 and 8 post-ovulation. AUC: Area under the Curve. This curve was then used to identify pregnant mares on days 7 and 8 post-ovulation. When analyzed using Youden's test, data showed that the uterine blood flow area in pregnant mares was greater than 1134 pixels on day 7 with a sensitivity of 69.1% and a specificity of 94.4% and an AUC: 0.836. After analysis, the cut off value for pregnant mares on day 8 post-ovulation was 912 pixels, with a sensitivity of 86.8 % and a specificity of 100% (AUC: 0.96). Consequently, evaluation of endometrial blood flow in pixels presented a greater predictive value on day 8 post-ovulation.

Table 1, Table 2, Table 3, Table 4, Table 5

**Table 1:** Descriptive statistics of endometrial blood flow in non-pregnant and pregnant mares expressed in pixels. Mean, standard deviation (SD), maximum, minimum and range.

	Non-pregnant mares					Pregnant mares				
	Mean	SD	Minimum	Maximum	Range	Mean	SD	Minimum	Maximun	Range
**Day 7**	727,67	260,32	250	1338	1088	1389,57	589,07	313	2819	2506
**Day 8**	508,36	220,35	119	904	785	1796,60	836,74	144	3998	3854

**Table 2:** Raw data file of uterine blood flow in non-pregnant mares on day 7. Nine barren mares were evaluated and 36 images (four images per mare) were assessed in the study. Pixel quantification was used to assess the degree of uterine vascular perfusion. The table shows the number of mares, the age of the mare, the uterine horn which was assessed and the respective value for blood flow in pixels. The mean, standard deviation (SD) and co-efficient of variation (CV) (%) between the two measurements from each uterine horn are also shown in the table.

Uterine Blood flow non-pregnant mare (Day 7)						
N° of mare	Age	Uterine horn	Blood flow pixel	Mean	SD	CV (%)
3	4	LEFT	400	455,00	77,78	17,09
		LEFT	510			
		RIGHT	250	388,50	195,87	50,42
		RIGHT	527			
21	4	LEFT	448	362,00	121,62	33,60
		LEFT	276			
		RIGHT	970	817,00	216,37	26,48
		RIGHT	664			
23	6	LEFT	466	647,00	255,97	39,56
		LEFT	828			
		RIGHT	948	988,00	56,57	5,73
		RIGHT	1028			
26	5	LEFT	307	598,00	411,54	68,82
		LEFT	889			
		RIGHT	627	673,00	65,05	9,67
		RIGHT	719			
29	2	LEFT	662	602,00	84,85	14,10
		LEFT	542			
		RIGHT	544	588,50	62,93	10,69
		RIGHT	633			
45	10	LEFT	786	701,00	120,21	17,15
		LEFT	616			
		RIGHT	1115	903,50	299,11	33,11
		RIGHT	692			
47	11	LEFT	993	1050,00	80,61	7,68
		LEFT	1107			
		RIGHT	1218	1278,00	84,85	6,64
		RIGHT	1338			
49	11	LEFT	814	864,00	70,71	8,18
		LEFT	914			
		RIGHT	865	829,00	50,91	6,14
		RIGHT	793			
51	9	LEFT	640	613,50	37,48	6,11
		LEFT	587			
		RIGHT	786	740,00	65,05	8,79
		RIGHT	694

**Table 3:** Raw data file of uterine blood flow in pregnant mares on day 7. Seventeen pregnant mares (positive flushing) were assessed and 68 images (four images per mare) were evaluated in the experiment. Pixel quantification was used to assess the degree of uterine vascular perfusion. The table shows the number of mares, the age of each mare, the uterine horn assessed and the values for blood flow in pixels. The mean, standard deviation (SD) and co-efficients of variation (CV) (%) between the two measurements from each uterine horn are also shown in the table.

Uterine Blood flow pregnant mare (Day 7)						
N° of mare	Age	Uterine horn	Blood flow pixel	Mean	SD	CV (%)
1	18	LEFT	1814	1656,50	222,74	13,45
		LEFT	1499			
		RIGHT	2618	2143,50	671,04	31,31
		RIGHT	1669			
4	6	LEFT	468	626,50	224,15	35,78
		LEFT	785			
		RIGHT	949	850,50	139,30	16,38
		RIGHT	752			
5	11	LEFT	1454	1447,50	9,19	0,64
		LEFT	1441			
		RIGHT	1204	1481,50	392,44	26,49
		RIGHT	1759			
6	11	LEFT	1853	1760,50	130,81	27,49
		LEFT	1668			
		RIGHT	1258	1607,00	493,56	28,49
		RIGHT	1956			
9	9	LEFT	2012	2057,00	63,64	3,09
		LEFT	2102			
		RIGHT	1578	1409,50	238,29	16,91
		RIGHT	1241			
11	7	LEFT	1182	1631,50	635,69	38,96
		LEFT	2081			
		RIGHT	1242	1476,00	330,93	22,42
		RIGHT	1710			
15	8	LEFT	1941	1936,50	6,36	0,33
		LEFT	1932			
		RIGHT	1982	2258,00	390,32	17,29
		RIGHT	2534			
16	7	LEFT	1912	1532,50	536,69	35,02
		LEFT	1153			
		RIGHT	1307	1568,00	369,11	23,54
		RIGHT	1829			
20	7	LEFT	2624	2103,00	736,81	35,04
		LEFT	1582			
		RIGHT	1900	1679,50	311,83	18,57
		RIGHT	1459			
22	6	LEFT	1799	1296,50	710,64	54,81
		LEFT	794			
		RIGHT	1391	945,00	630,74	66,74
		RIGHT	499			
24	5	LEFT	1258	1291,50	47,38	3,67
		LEFT	1325			
		RIGHT	776	859,00	117,38	13,66
		RIGHT	942			
25	5	LEFT	1478	1455,50	31,82	2,19
		LEFT	1433			
		RIGHT	858	1387,00	748,12	53,94
		RIGHT	1916			
33	3	LEFT	746	1365,00	875,40	64,13
		LEFT	1984			
		RIGHT	903	778,00	176,78	22,72
		RIGHT	653			
34	2	LEFT	1412	1289,00	173,95	13,49
		LEFT	1166			
		RIGHT	1402	1482,00	113,14	7,63
		RIGHT	1562			
35	2	LEFT	494	403,50	127,99	31,72
		LEFT	313			
		RIGHT	655	827,50	243,95	29,48
		RIGHT	1000			
36	2	LEFT	727	611,50	163,34	26,71
		LEFT	496			
		RIGHT	820	645,00	247,49	38,37
		RIGHT	470			
41	6	LEFT	2045	1611,50	613,06	38,04
		LEFT	1178			
		RIGHT	1326	1062,50	372,65	35,07
		RIGHT	799

**Table 4:** Raw data file showing uterine blood flow in non-pregnant mares on day 8. Nine barren mares were evaluated and 36 images (four images per mare) were assessed in the study. Pixel quantification was used to assess the degree of uterine vascular perfusion. The table shows the number of mares, the age of each mare, the uterine horn assessed and the values for blood flow in pixels. The mean, standard deviation (SD) and co-efficients of variation (CV) (%) between the two measurements from each uterine horn are also shown in the table.

Uterine Blood flow non-pregnant mare (Day 8)						
N° of mare	Age	Uterine horn	Blood flow pixel	Mean	SD	CV (%)
2	6	LEFT	140	152,50	17,68	11,59
		LEFT	165			
		RIGHT	510	325,00	261,63	80,50
		RIGHT	140			
17	7	LEFT	292	328,00	50,91	15,52
		LEFT	364			
		RIGHT	284	337,00	74,95	22,24
		RIGHT	390			
38	3	LEFT	595	536,50	82,73	15,42
		LEFT	478			
		RIGHT	369	337,00	45,25	13,43
		RIGHT	305			
40	3	LEFT	563	341,00	313,96	92,07
		LEFT	119			
		RIGHT	317	323,00	8,49	2,63
		RIGHT	329			
43	3	LEFT	490	440,50	70,00	15,89
		LEFT	391			
		RIGHT	427	398,00	41,01	10,30
		RIGHT	369			
46	10	LEFT	686	713,00	38,18	5,36
		LEFT	740			
		RIGHT	614	647,00	46,67	7,21
		RIGHT	680			
48	11	LEFT	889	783,50	149,20	19,04
		LEFT	678			
		RIGHT	904	728,50	248,19	34,07
		RIGHT	553			
50	11	LEFT	729	699,50	41,72	5,96
		LEFT	670			
		RIGHT	473	673,50	283,55	42,10
		RIGHT	874			
52	9	LEFT	544	670,00	178,19	26,60
		LEFT	796			
		RIGHT	660	717,00	80,61	11,24
		RIGHT	774

**Table 5:** Raw data file showing uterine blood flow in pregnant mares on day 8. Seventeen pregnant mares (positive flushings) were assessed and 68 images (four images per mare) were evaluated in the study. Pixel quantification was used to assess the degree of uterine vascular perfusion. The table shows the number of mares, the age of each mare, the uterine horn assessed and the values for blood flow in pixels. The mean, standard deviation (SD) and co-efficients of variation (CV) (%) between the two measurements from each uterine horn are also shown in the table.

Uterine Blood flow non-pregnant mare (Day 8)						
N° of mare	Age	Uterine horn	Blood flow pixel	Mean	SD	CV (%)
2	6	LEFT	140	152,50	17,68	11,59
		LEFT	165			
		RIGHT	510	325,00	261,63	80,50
		RIGHT	140			
17	7	LEFT	292	328,00	50,91	15,52
		LEFT	364			
		RIGHT	284	337,00	74,95	22,24
		RIGHT	390			
38	3	LEFT	595	536,50	82,73	15,42
		LEFT	478			
		RIGHT	369	337,00	45,25	13,43
		RIGHT	305			
40	3	LEFT	563	341,00	313,96	92,07
		LEFT	119			
		RIGHT	317	323,00	8,49	2,63
		RIGHT	329			
43	3	LEFT	490	440,50	70,00	15,89
		LEFT	391			
		RIGHT	427	398,00	41,01	10,30
		RIGHT	369			
46	10	LEFT	686	713,00	38,18	5,36
		LEFT	740			
		RIGHT	614	647,00	46,67	7,21
		RIGHT	680			
48	11	LEFT	889	783,50	149,20	19,04
		LEFT	678			
		RIGHT	904	728,50	248,19	34,07
		RIGHT	553			
50	11	LEFT	729	699,50	41,72	5,96
		LEFT	670			
		RIGHT	473	673,50	283,55	42,10
		RIGHT	874			
52	9	LEFT	544	670,00	178,19	26,60
		LEFT	796			
		RIGHT	660	717,00	80,61	11,24
		RIGHT	774			

## 2 Experimental Design, Materials, and Methods

The aim of this experiment was to assess whether evaluation of endometrial blood flow in mares could be rated as a good tool for identification of early pregnancy. In total, fifty-two mares of a range of different ages (2-18 years old) were used to obtain this data. Ultrasonographic examination with Power Doppler was used to assess uterine blood flow prior to uterine flushing procedures for embryo recovery on both days 7 and 8 after ovulation. Two transverse images were captured per uterine horn and analysis of images was subsequently performed at a later date using Image J 1.48 software.

Prior to ultrasonographic examination all mares were sedated with romifidine (0.02 mg/kg) (Sedivet® Boehringer Ingelheim, Barcelona, España). MyLabFive Vet equipment (Esaote S.p.A, Genova, Italy) with a linear 5-7.5 MHz probe was used to complete the assessment. The specific settings used were: gain: 70%, PRF: 1,4 KHz at a depth of 9 cm. Examination was performed per rectum and the ultrasound probe was placed transversely at a mid-point of both uterine horns in order to capture two still images per horn. Signals indicating blood flow in all the endometrial, myometrial and perimetrial vessels were visualized using Power flow modes due to their capacity for greater sensitivity for detection of weak signals from small vessels. Degree of uterine vascular perfusion was objectively assessed using quantification of pixels in bitmap (BMP) format. Blind analysis was performed on a total of 208 images (four images per mare). Subsequent computer analysis of Doppler images was executed using ImageJ v1.48 software (National Institute of Health, USA). User-defined selections and intensity-thresholded objects can be analyzed with ImageJ, which is a Java-based image processing program. Analysis of endometrial vascular perfusion was performed using spot meter techniques, measuring blood flow area. A color threshold restricted to the uterine horn in question was applied first for blood vessel detection. Following this, the total area for the region of interest (blood vessels) was calculated.

## Author Contribution Section

PNO: Conceptualization, conceived the study and performed experiments, methodology and data curation; FEMC: performed the analysis of data with Image J (Software), validation and reviewed the draft. GGP: data curation and English review (native speaker) writing - review & editing, Software. JMO: Methodology, data curation. FJP: Conceptualization, funding acquisition, English review and statistical analysis, COF: Conceptualization, conceived the study, supervision, formal analysis and roles/writing - original draft.

## Competing Interests

The authors declare that they have no known competing financial interests or personal relationships which have, or could be perceived to have, influenced the work reported in this article.
